# KRAB zinc finger protein ZNF676 controls the transcriptional influence of LTR12-related endogenous retrovirus sequences

**DOI:** 10.1186/s13100-021-00260-0

**Published:** 2022-01-18

**Authors:** Alexandra Iouranova, Delphine Grun, Tamara Rossy, Julien Duc, Alexandre Coudray, Michael Imbeault, Jonas de Tribolet-Hardy, Priscilla Turelli, Alexandre Persat, Didier Trono

**Affiliations:** 1 School of Life Sciences, EPFL, Lausanne, Switzerland; 2 Department of Genetics, University of Cambridge, Cambridge, UK

## Abstract

**Background:**

Transposable element-embedded regulatory sequences (TEeRS) and their KRAB-containing zinc finger protein (KZFP) controllers are increasingly recognized as modulators of gene expression. We aim to characterize the contribution of this system to gene regulation in early human development and germ cells.

**Results:**

Here, after studying genes driven by the long terminal repeat (LTR) of endogenous retroviruses, we identify the ape-restricted ZNF676 as the sequence-specific repressor of a subset of contemporary LTR12 integrants responsible for a large fraction of transpochimeric gene transcripts (TcGTs) generated during human early embryogenesis. We go on to reveal that the binding of this KZFP correlates with the epigenetic marking of these TEeRS in the germline, and is crucial to the control of genes involved in ciliogenesis/flagellogenesis, a biological process that dates back to the last common ancestor of eukaryotes.

**Conclusion:**

These results illustrate how KZFPs and their TE targets contribute to the evolutionary turnover of transcription networks and participate in the transgenerational inheritance of epigenetic traits.

**Supplementary Information:**

The online version contains supplementary material available at 10.1186/s13100-021-00260-0.

## Introduction

Transposable element (TE)-derived sequences account for more than half of the human genome, with over 4.5 million recognizable integrants. Most are incapable of further spread due to inactivating mutations but many can still influence genome expression. TE-embedded regulatory sequences (TEeRS) are important repositories of transcription factor binding sites, and can act as promoters, enhancers, insulators, as producers of small interfering RNAs, microRNAs and long non-coding RNAs, or as sites of nucleation for heterochromatin [6, 10, 24, 74]. Accordingly, TEeRS impact gene expression in both physiological and pathological circumstances, as exemplified on the one hand by the long terminal repeats (LTRs) of primate-specific endogenous retroviruses (ERVs) governing expression of the amylase and prolactin genes in human salivary glands and placenta, respectively [17, 81], and on the other hand by the detection of TE-driven oncogene-encoding transcripts in many human cancers [37].

TEeRS play a particularly prominent role during gametogenesis and early embryogenesis, as the widespread genome reprogramming that characterizes these developmental periods erases epigenetic marks normally responsible for silencing TEs. Illustrating the general biological relevance of this phenomenon, hominoid-specific TE-based enhancers foster the opening of chromatin during human spermatogenesis and zygotic genome activation (ZGA) [48, 62]. Adding to these genome-wide influences, numerous TEeRS-driven gene transcripts, or transpochimeric gene transcripts (TcGTs), are detected both in germ cells and in early embryos [5, 7, 23, 27], suggesting that TEs exert gene-specific effects in these settings.

KRAB-containing zinc finger proteins (KZFPs) are major controllers of TEeRS activity. *KZFP* genes emerged some 420 million years ago in the last common ancestor of tetrapods, coelacanth and lungfish, and have since been then subjected to a high evolutionary turnover, with 30 and 90% of the ~360 human *KZFP* genes primate- and placental mammal-restricted, respectively [16, 18, 35, 47, 57]. KZFPs are characterized by an N-terminal KRAB domain and a C-terminal poly-zinc finger array endowed with sequence-specific DNA binding potential. For a large majority of human family members, the first of these functional domains governs the recruitment of a KAP1/TRIM28-nucleated heterochromatin-inducing complex, and the second docking at specific subgroups of TE integrants [18, 30, 35, 53, 56, 58, 86]. Recent evidence points to the importance of KZFP-mediated taming of TEeRS in the immediate aftermath of zygotic genome activation and in modulating gene expression in embryonic stem cells and in organs such as the brain [20, 35, 36, 39, 62, 83, 86]. The present study was undertaken to examine whether KZFPs and TEeRS partner up to shape the transcriptional landscape during germline formation and the transmission of epigenetic cues from germ cells to early embryos. It led to the identification of ZNF676 as a hominoid-specific KZFP controlling the transcriptional activity of a subset of ERV-embedded regulatory sequences active during gametogenesis and during the first few divisions following fertilization of the egg, and influencing the transgenerational inheritance of epigenetic marks at these loci.

## Results

### LTR12-based promoters are major TcGT drivers in human gametes and early embryos

**Fig. 1:**
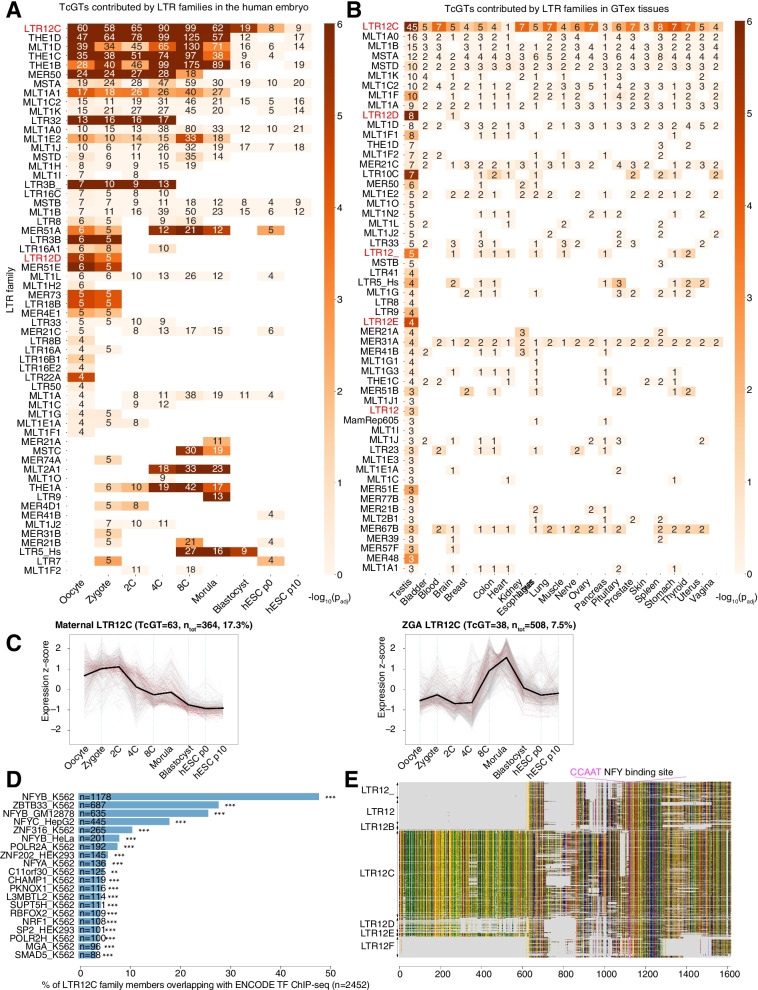
LTR12/ERV9 elements are expressed in early embryonic development and contribute new TSS to genes expressed before and after ZGA. **A** Number of detected LTR-driven transpochimeric gene transcripts (TcGTs) in a de novo transcriptome assembly of human embryonic development RNA-seq [88]. Number represents the transcripts found (TSS overlapping with LTR, minimum 1 read spanning junction), heatmap color represents -log10(padj). The p-value was computed through a hypergeometric test and adjusted for multiple testing. **B** Number of detected LTR-driven TcGTs in a de novo transcriptome assembly of a subset of tissues from the GTex dataset [50], method same as in (**A**). **C** Z-score clusters of expression of all detected LTR12C loci over human embryonic development stages [88]. 2 clusters were formed. Red lines = LTR12C loci with at least one detected TcGT at any stage of development in (**A**). Grey lines = LTR12C loci without known TcGTs. Thick black line: mean expression value across replicates for each sample. **D** Transcription factors from ENCODE with ChIP-seq peaks overlapping LTR12C loci with minimum overlap of 1 bp. *: *p*<0.05, **: *p*<0.01, ***: *p*<0.001, Fisher hypergeometric test, adjusted for multiple testing. **E** Multiple sequence alignment of all LTR12 family members (Aliview colors for DNA bases, grey = gap) with conserved NFY motif highlighted

We searched for TcGTs in a single-cell RNA-seq dataset generated from human early embryos [88], focusing on transcripts driven from the long terminal repeat (LTR) of endogenous retroviruses (ERVs) because these are notorious contributors of alternative gene promoters [23, 59]. For this, we selected RNAs with an LTR-encoded 5’-end with a minimum of one downstream read splicing into an annotated gene. We found that TcGTs were generally driven by so-called solo LTRs, which are ERV internal recombination products devoid of coding sequences [2], rather than by their full-length proviral counterparts. LTR12C, which is present at some 2,500 copies in the human genome and belongs to an LTR subgroup normally associated with ERV9 internal sequences, topped the list of TcGT-driving ERV promoters in the oocyte (Fig. 1A, Supplementary Table 1). Also standing out were the MaLR-derived THE1D, MLT1D, THE1C and THE1B LTRs, also previously identified as prominent TcGT drivers in mouse early embryos [7, 59]. In this single-cell RNA-seq dataset, LTR12C-promoted TcGTs were detected in oocytes, which was confirmed by analyzing an oocyte transcriptome independently obtained by bulk sequencing [7, 31] (Fig. S1A, Supplementary Table 1). Both our observations of MaLR and LTR12C TcGT profiles confirm prior findings made in the oocyte [7], while LTR12C has also been shown to initiate transcription in other contexts [4, 5]. Upon analyzing RNA-seq data from the GTEx consortium [50], we notably found LTR12C to be also the most frequent source of LTR-driven TcGTs in the testis (Fig. 1B, Supplementary Table 3).

We then asked whether individual LTR12C integrants differed in transcriptional behavior. We could delineate two clusters by examining early embryonic RNA-seq data (Fig. 1C). The first, defined as maternal cluster, comprised 364 LTR12C loci, including 63 TcGT-driving LTRs, from which transcripts were detected in oocyte but dropped from the 2-cell (2C) stage on. The second, coined ZGA cluster, encompassed 508 loci, including the promoters of 38 TcGTs, that were expressed from the 8-cell (8C) up to the morula stage. When this census was expanded to all expressed LTR12/ERV9 loci, the same bimodal distribution was observed, and LTR12C was confirmed as the main contributor to both ERV-derived RNA reads and TcGT production in either cluster (Fig. S1B). TcGTs were both observed as alternative promoters and as truncated transcripts (Fig. S1C).

### LTR12/ERV9 TEeRS are repressed by ZNF676/ZNF728

We sought to identify transcription factors responsible for regulating the transcription of TcGT-driving LTR12C and other LTR12/ERV9 integrants. Upon examining the ENCODE dataset [78], we identified NFYB as the most frequent high-affinity ligand of LTR12 (Fig. 1D), consistent with the proposal that a complex formed by NFY-A, -B and -C is required to activate these retroviral promoters [43]. The NF-Y binding motif (CCAAT) was strongly conserved across all 7 LTR12 subfamilies (Fig. 1E). However, we also noticed that LTR12C (*n* = 2453), LTR12D (*n* = 377) and LTR12E (*n* = 104) integrants displayed a ~600bp-long 5’ extension (Fig. 1E). Since this proximal appendix coincided with their acting as TcGT drivers (Fig. 1A, Fig. 1B, Fig. S1A), we reasoned that it might have facilitated their cooption as gene promoters.

**Fig. 2:**
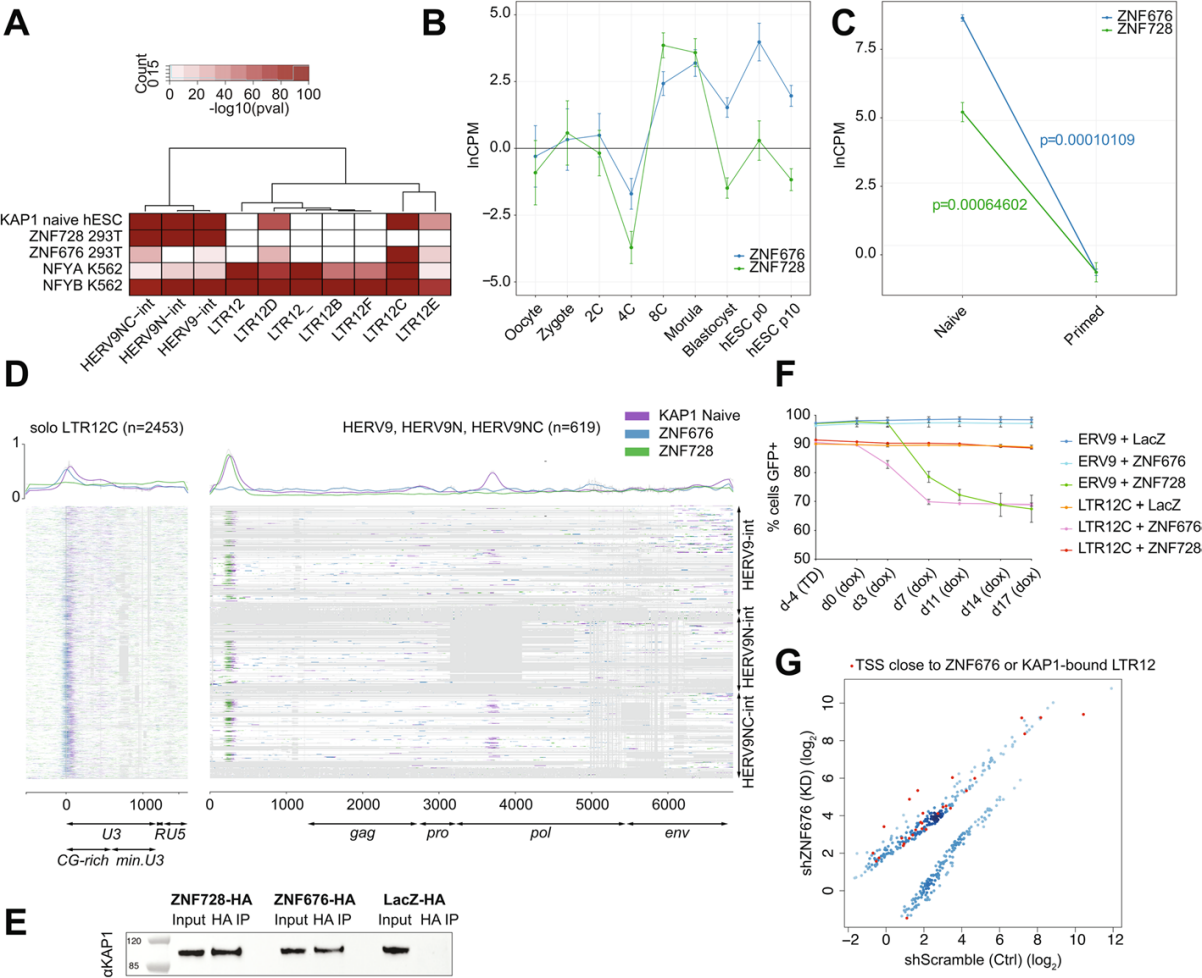
ZNF676 and ZNF728 target respectively LTR12 and ERV9 elements and are able to repress their TE targets through the recruitment of KAP1. **A** Heatmap displaying KAP1, NFYA/B ChIP-seq & KZFP ChIP-exo enrichment over LTR12/ERV9 family members. KZFP ChIP-exo in HEK293T. NFYA/B ChIP-seq in K562 cells from ENCODE. KAP1 ChIP-seq in naive hESCs from [79]. p-value obtained by binomial test, corrected for TE subfamily size. **B** *ZNF676* and *ZNF728* averaged expression across embryonic development, RNA-seq from [88]. Error bars represent biological replicates. **C** *ZNF676* and *ZNF728* expression in reset to naive and primed WIBR3 hESCs, RNA-seq from [80]. **D** Multiple sequence alignments of LTR12C loci and 3 ERV9 subfamilies (gaps = grey, aligned sequences = white) overlaid with ZNF676-HA and ZNF728-HA in HEK293T cells and KAP1 in naive hESC ChIP-seq signals (colored). Proviral genomic features of ERV9 elements as detected by RetroTector, features of LTR12C as described in [49]. **E** HA immunoprecipitation of stably expressed ZNF676-HA and ZNF728-HA in K562 cells, followed by western blot detection of KAP1. **F** GFP fluorescence intensity across time in repression assay experiments (biological replicates, *n*=2). TE loci cloned upstream of PGK-GFP cassette are co-transduced with ZNF676, ZNF728 or LacZ with subsequent doxycycline-mediated induction at d0. **G** Dot plot representing (in red) ZNF676 and KAP1 targets among genes deregulated upon ZNF676 shRNA-mediated knockdown in Win1 naive embryonic stem cells

We previously proposed that KZFPs foster the domestication of TEeRS by taming their regulatory potential [15, 62, 82]. We thus asked which members of the family were responsible for the transcriptional control of LTR12 integrants. Complementing a previously published ChIP-exo-based catalogue of human KZFP genomic targets [35] through additional studies, we identified ZNF676 as enriched over the 5’ extension of LTR12C, D and E solo-LTRs and ZNF728 over the 5’ untranslated region of ERV9-int sequences (Fig. 2A, Fig. S2A). We confirmed these results by performing further ChIP-seq experiments in HEK293T and H1 hESC overexpressing HA-tagged forms of *ZNF676* and *ZNF728* (Fig. S2B). Of note, TcGT-driving LTR12C units were targeted by ZNF676 but not ZNF728, and LTR12/HERV9 integrants recognized by either KZFP were often bound by NFYA/NFYB in K562 cells (Fig. 2A).

We went on to verify that these two KZFPs could act as transcriptional repressors. First, we observed that the subsets of TEs bound by ZNF676 or ZNF728 recruited KAP1 in naive embryonic stem cells (hESC) (Fig. 2A, Fig. S2A), and that subfamily enrichment in H1 hESCs was very similar to that observed in HEK293T cells (Fig. S2B). A consensus derived from three independent ChIP-seq experiments performed in HEK293T had a large overlap with KAP1 (Fig. S2B, 425 LTR12C loci with ZNF676 binding out of 492 also presented KAP1 peaks in naive hESC) and was therefore used to infer high-confidence ZNF676 binding sites for the rest of this study. Correspondingly, transcript levels of *ZNF676* and *ZNF728* were lowly abundant in oocyte and zygote, increased during ZGA and dropped in blastocysts (Fig. 2B), and both *KZFP*s were markedly more expressed in naïve than in primed hESCs (Fig. 2C, Fig. S2C).

We also observed that KAP1 was enriched in naive hESCs over both the 5’extension of LTR12C and a short appendage found upstream of the predicted primer-binding site of ERV9-int integrants, corresponding to the exact respective binding sites of ZNF676 and ZNF728 (Fig. 2D). We corrected the annotated ZNF676 cDNA (Fig. S2D, E), wrongly predicted to encode for a protein with an N-terminally truncated KRAB A-box due to a missed exon, and then verified that both ZNF676 and ZNF728 could be co-immunoprecipitated with KAP1 (Fig. 2E). Furthermore, overexpression of either KZFP in HEK293T cells resulted in the silencing of lentivector-borne PGK-GFP reporter cassettes juxtaposed to its cognate LTR12- or HERV9-derived target (Fig. 2F, Fig. S2F).

Upon depleting *ZNF676* in naive hESC by lentivector-mediated RNA interference, we observed the dysregulation of 425 genes, 29 of them located within 50kb of an LTR12C locus enriched for ZNF676 or KAP1 in respectively HEK293T or naïve hESCs (Fig. 2G), which was more than expected by chance (Fisher test, *p*-value = 0.012). We concluded that the regulatory activity of affected LTR12C elements included enhancer functions, as previously reported [13, 33, 49, 61, 89]. More generally, there was a highly significant enrichment for KAP1-bound LTR12C integrants within 50kb of the TSS of upregulated genes (Fisher test, adjusted p-value = 1.6E-12) (Fig. S2G). This supports a model whereby ZNF676 controls the expression of genes via its repression of LTR12C-based TEeRS.

### ZNF676 and its LTR12 targets are hominid-specific

**Fig. 3:**
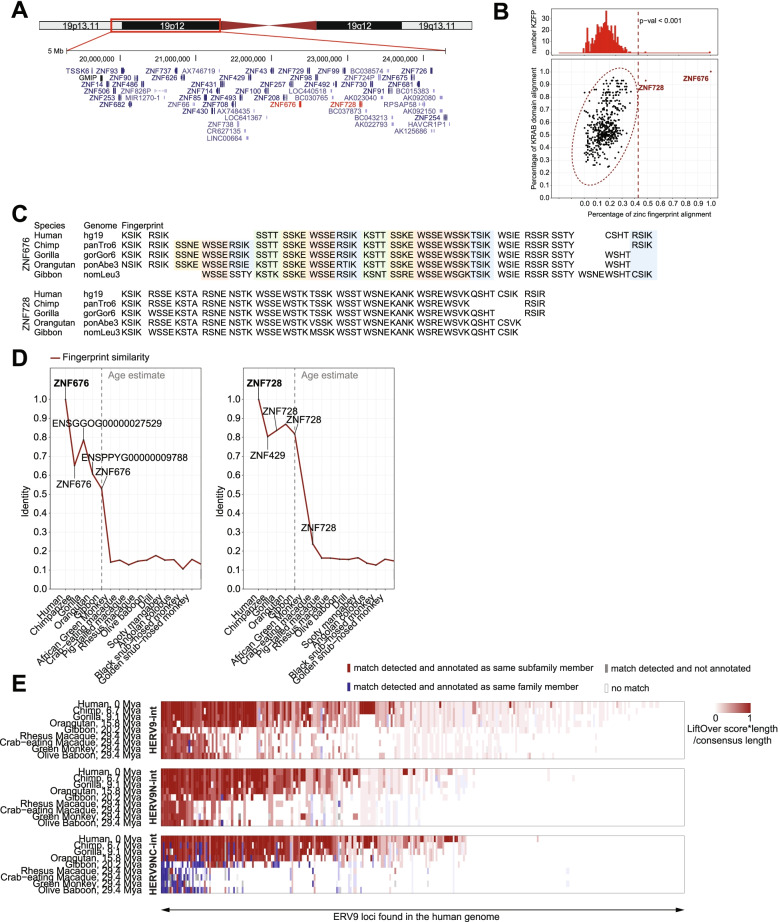
ZNF676 and ZNF728 are evolutionary paralogs that target respectively LTR12 and ERV9 families, and have appeared ~20MYA, concomitant with their target TEs. **A** Annotated gene transcripts in KZFP gene cluster containing *ZNF676* and *ZNF728* on chr19, hg19. Data from “UCSC Genes” track in UCSC genome browser. **B** Comparative protein alignment of ZNF676’s and ZNF728’s KRAB zinc fingerprints (4 DNA-contacting residues per zinc finger, X axis) and KRAB domains (Y axis). Closeness to 1 indicates high similarity. **C** Comparative alignment of ZNF676’s and ZNF728’s DNA-contacting residues (4 per zinc finger, positions -1, 2, 3, and 6) across primate genomes. Similar zinc fingers within ZNF676 are highlighted in the same color. **D** Primate-specificity of ZNF676 and ZNF728 orthologs. X-axis: genomes scanned by in-house KZFP-detecting program. Red line: similarity of DNA-contacting residues in the closest ortholog across species. Grey line: estimated appearance date of ZNF676 in primate genomes. Visual estimate based on sharp increase of the DNA-binding domain similarity. **E** Colonization of primate genomes by full-length ERV9 integrants. Heatmap depicting all Repeatmasker-annotated ERV9 loci in hg38 human genome and their liftOver orthologous loci in primate species. White = not detected. Red = detected and annotated as same subfamily member. Blue = detected and annotated as same family member. Grey = detected and not annotated. Color intensity = similarity (by percentage of sequence aligned) normalized by integrant length relative to consensus

*ZNF676* and *ZNF728* are located within a rapidly evolving *KZFP* gene cluster on chromosome 19 (Fig. 3A). Although separated by >700 kb and four other *KZFP* genes, they encode for two highly related proteins, with an amino-acid alignment of ZNF676 poly-zinc finger and KRAB domains with the corresponding regions of all other human KZFPs pointing to ZNF728 as its closest paralog (Fig. 3B). The zinc fingerprints of the two proteins, that is, the quadruplets of amino acids within their zinc fingers predicted to determine the sequence specificity of their DNA binding [87], notably display similarly repetitive configurations, likely the consequence of internal duplications (Fig. 3C) with acquisition of significantly diverged binding sites. By searching for orthologs in other species, we traced *ZNF676* and *ZNF728* to the last common ancestor of human, chimpanzee, gorilla, orangutan and gibbon, (Fig. 3C, D), and did not find any earlier orthologs. Therefore, *ZNF676* and *ZNF728* are ape-restricted *KZFP* genes that emerged some 20 million years ago (mya). In parallel, we documented the presence or HERV9, HERV9N and HERV9NC integrants in the genomes of all great apes, with at least some HERV9 and HERV9N inserts additionally found in rhesus and crab-eating macaques. Our analysis indicated that the ERV9 family of retroviruses invaded the human ancestral genome more than 30 mya (Fig. 3E), in line with earlier estimates [11, 51]. However, LTR12C sequences, the main TE targets of ZNF676, while observed in very small numbers in the olive baboon genome, underwent their main expansion in the genomes of Hominidae (Fig. S3A). Thus, illustrating a trend observed for many *KZFP*s [35, 57], *ZNF676* and *ZNF728* have roughly the same evolutionary ages as their TE targets, suggesting coevolution.

### KZFP-modulated differential LTR12C methylation in the germline

In the first few days of embryogenesis, the KZFP/KAP1 system can trigger the DNA methylation of its genomic targets [85]. Relatedly, ZFP57 and ZNF445 are responsible for the maintenance of imprinting marks during this period of profound epigenetic remodeling [52, 63, 64, 73, 75]. Erasure of DNA methylation similarly occurs in early human primordial germ cells (hPGCs) at ~7-9 weeks of development, and this reprogramming event is a prerequisite for the establishment of a pluripotent zygote [26]. However, around 8% CpG dinucleotides remain methylated, many of which reside within hominoid-specific TE integrants [77]. It was recently suggested that KZFPs may contribute to protecting these sequences from demethylation and that such hPGC-methylated TEeRS may then be marked to function as enhancers in developing and adult organs [14].

**Fig. 4:**
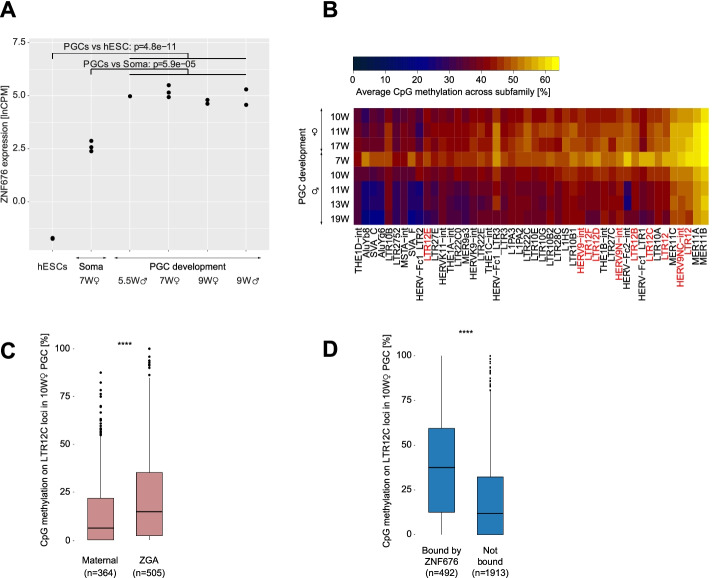
KZFPs such as ZNF676 are candidates for protecting LTR12C elements against genome-wide demethylation in human PGC development. **A** *ZNF676* expression across hPGC development. RNA-seq data from [77]. **B** Heatmap depicting average DNA methylation levels per TE subfamily across male and female hPGC development. RRBS data from Guo et al. [28]. Most methylated TE families represented. **C** Boxplots representing RRBS DNA methylation levels in female hPGC at week 10 of development across LTR12C loci known to belong to the maternal or ZGA clusters. Wilcoxon test for significance. **D** Boxplots representing RRBS DNA methylation levels in female hPGC at week 10 of development across LTR12C loci identified as ChIP-seq targets or non-targets of ZNF676 (consensus of 3 replicates in HEK293T cells). Wilcoxon test for significance

To explore this hypothesis, we examined *ZNF676* expression in the germline, and found that it was highly expressed in male and female PGCs at all profiled timepoints, consistent with a role not only in the earliest days of embryogenesis but also during the establishment of the germline (Fig. 4A, Fig. S4A). *ZNF676* expression was also detected in adult testes (Fig. S4B), suggesting that it is retained during male gametogenesis. Upon profiling the DNA methylation status of TEs in PGCs, we confirmed that integrants from all LTR12/ERV9 subfamilies retain a high level of CpG methylation in this setting, despite the genome-wide erasure of this mark characteristic of this developmental stage [28, 77] (Fig. 4B). This behavior is comparable to that of murine IAP elements, which also remain methylated in mouse PGCs [69]. This led us to conduct a closer investigation of the LTR12C subset, comparing the 10-week female PGC DNA methylation levels of integrants assigned to either the maternal cluster or the ZGA cluster based on their mode of early embryonic expression (Fig. 1C). The results indicated that LTR12C integrants expressed as part of the ZGA cluster displayed statistically significant higher levels of DNA methylation in PGCs than LTR12C integrants belonging to the maternal cluster (Fig. 4C), as were LTR12C integrants recognized by ZNF676 compared to the ones not bound by this KZFP (Fig. 4D). This was consistent with the observation that a higher proportion of loci from the ZGA than from the maternal cluster were targeted by ZNF676 (Fig. S4C). These data indicate that ZNF676 binding coincides with the protection of at least some LTR12C integrants from demethylation in the germline, with a possible influence on their transcriptional fate in the nascent embryo. As such, this supports the hypothesis that KZFPs participate in the establishment of epigenetic marks that are transmitted across generations [14, 77].

### ZNF676/LTR12C control genes important for ciliogenesis

**Fig. 5:**
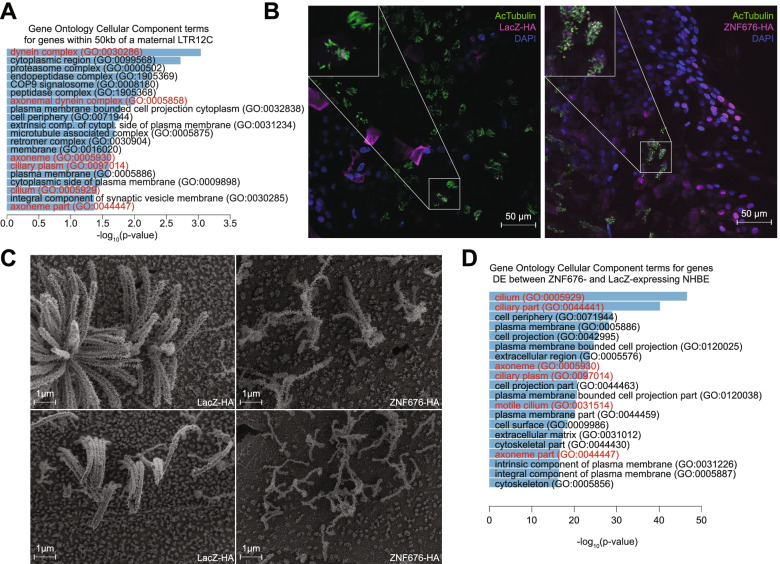
ZNF676 regulates several germline genes involved in cilia motility, and its overexpression is able to disrupt ciliogenesis. **A** Gene Ontology Cellular Component terms (GOstats R package, hypergeometric test, non-adjusted p-value) for which genes within 50 kb of an LTR12C assigned to the maternal cluster were enriched. **B** Immunofluorescence staining of normal human bronchoepithelial cells (Lonza) overexpressing HA-tagged LacZ (control) or ZNF676, differentiated into ciliated cells (4 weeks at air-liquid interface). Blue: DAPI (nuclei), green: acetylated tubulin (cilia), purple: HA-tagged lentivector-mediated overexpression of protein of interest (left, LacZ, right, ZNF676). **C** Representative scanning electron microscopy images of the surface of Au/Pd-coated primary human bronchoepithelial cells (Lonza) differentiated into ciliated cells (4 weeks at air-liquid interface). Magnification: 30’000x. Left: lentivector-mediated overexpression of LacZ (control). Right: lentivector-mediated overexpression of ZNF676. **D** Gene Ontology Cellular Compartment terms enriched among differentially expressed genes in an RNA-seq of ZNF676-overexpressing NHBE cells compared to GFP-overexpressing ones

It remained that a number of LTR12C loci, including ones driving TcGTs, became demethylated in PGCs and were transcriptionally active in oocyte and zygote as part of the maternal cluster of expressed LTR12C inserts. A gene ontology analysis of this maternal cluster indicated that genetic units located within 50kb of these integrants were enriched for terms related to the dynein complex (Fig. 5A, Supplementary Table 2), suggesting that they influenced the expression of this functional group. An identical analysis performed on genes within 50kb of LTR12C loci detected in testis also pointed to dynein-related events (Fig. S5A, Supplementary Table 3). The proper expression of ciliary dyneins is essential for sperm flagellum assembly and motility, which in turn plays a crucial role in male fertility. Consistent with this finding, LTR12C, D and E integrants were previously found to become accessible in human spermatogonial stem cells [29] and subsequently expressed in prospermatogonia and prospermatogonia-like models [34], suggesting that they might regulate genes involved in sperm development. Supporting a role for ZNF676/LTR12-mediated regulation of this process, *ZNF676*-depleted naïve hESC displayed increased levels of transcripts encoding the dynein DNAH11 and the protein C4orf47 (Fig. S5B). C4orf47 is a highly conserved protein present in spermatid flagella and in cilia of the fallopian tube and respiratory tract ([22, 84], Human Protein Atlas available from http://www.proteinatlas.org), and we could detect DNAH11 in the proximal region of respiratory cilia by immunohistochemistry staining (Fig. S5C). A strong ZNF676 peak was detected in the regulatory regions of both genes, although an underlying LTR12C sequence was readily identifiable only for *DNAH11* (Fig. S5B). These results suggested a model whereby LTR12C-based TEeRS promote ciliogenesis during gamete formation and ZNF676 mediates the early embryonic downregulation of genes participating in this process (Fig. S5D).

Given the lack of experimental system to study human spermatogenesis, we took advantage of similarities between motile airway cilia and sperm flagellum. We transduced primary normal human broncho-epithelial (NHBE) cells with lentivectors harboring dox-controllable versions of *ZNF676* or *LacZ* and induced the expression of these transgenes upon triggering differentiation into ciliated cells at an air-liquid interface. Both immunofluorescence enhanced microscopy and scanning electron microscopy revealed profound perturbations of ciliogenesis in *ZNF676*-overexpressing cells, characterized by abnormally short and disorganized cilia (Fig. 5B, C). Furthermore, a comparison of the transcriptomes of these cells and their *LacZ*-transduced controls revealed that a large fraction of differentially expressed genes were related to the cilium (Fig. 5D, Supplementary Table 4).

## Discussion

This work reveals a connection between a KRAB-containing zinc finger protein and the epigenetic marking of TE-embedded regulatory sequences in the human germline and the control of their transcriptional activity during gametogenesis and early embryogenesis. It also illustrates how TEs and their KZFP controllers participate in the rapid evolutionary turnover of transcriptional networks, by demonstrating that ciliogenesis, a biological process that dates back to the last common ancestor of eukaryotes more than one billion years ago [67], is influenced in human by the coordinated action of *cis*- and *trans*-regulators that only appeared with the first hominids, that is, less than 20 million years ago.

It was long established that the KRAB-mediated recruitment of KAP1 to genomic loci can trigger their de novo methylation if it occurs during the first few days of embryogenesis [85]. It was also demonstrated that ZNF445 and ZFP57 are responsible for the maintenance of imprints during human and murine early embryonic development [52, 64, 75], with possibly some contribution from ZNF202 in human [55] and ZFP708 in mouse [68]. Our results indicate that a KZFP binding correlates with the maintenance of DNA methylation during gametogenesis, with ZNF676 targeting a subset of LTR12 loci that is protected from demethylation in PGCs. Therefore, the KZFP/KAP1 system plays a dual role in allowing for the trans-generational inheritance of DNA methylation, protecting specific loci from its erasure during the genome-wide reprogramming that takes place either in the germline (e.g. for ZNF676 at LTR12C loci) or in early embryo (e.g. for ZNF445 and ZFP57 at imprinted loci).

HERV9-derived LTR12C-embedded regulatory sequences are recent invaders of the human ancestral genome, yet we find them to govern biological processes essential to reproduction and physiology, as they influence the expression of genes important for the formation of the sperm flagellum and respiratory cilia. LTR12/ERV9 integrants can be traced back at the earliest to the last common ancestor of human and macaque, some 30 mya. However, the 5’ appendage that characterizes LTR12C/D/E is almost exclusively found in hominoids, indicating that it expanded about ten million years later, at the same time as its ZNF676 ligand. Although devoid of any component fundamentally essential for retroviral propagation, this 5’ extension was conserved whilst its ERV carriers kept spreading in the genomes of their hosts. Thus, even though ZNF676 is a transcriptional repressor, it not only did not prevent but likely fostered the expansion of its LTR12C-driven ERV targets. We propose that ZNF676 facilitated LTR12C co-option by ensuring that its transcriptional activity was restricted to critical developmental stages or specific tissues. ZNF676 and ZNF728 display another interesting evolutionary interplay, as they are endowed with resembling zinc fingerprints yet bind to regions of LTR12/HERV9 that are not only spatially distinct but also harbored today by dissociated sets of integrants, namely solo LTR12 for the former and HERV9-int for the latter.

The NF-Y complex was previously found to participate in the activation of LTR12 elements, most of which bear its CCAAT binding motif [9, 43, 89]. Our identification of KZFPs targeting a subset of LTR12/ERV9 integrants hints at opposing influences between NF-Y-induced activation and KAP1-mediated repression wherever these KZFPs are expressed. ERVs bearing binding sites for KZFPs such as ZNF676 might therefore have been advantaged in their colonization of the germline, as they provided promoters that could be strongly activated by NF-Y yet timely repressed through KAP1 recruitment, as illustrated here with the LTR12C-regulated and ZNF676-controlled dynein-related genes. Initially proposed by Britten and Davidson some fifty years ago [8], the coordinated influence of TE-borne *cis*-acting sequences on regulomes implies a high degree of evolutionary turnover. This is illustrated by the orchestration in human of biological events as highly conserved as flagellum formation and ciliogenesis by the concerted action of LTR12C and ZNF676, both of which have recently appeared in the primate lineage.

Our results also have potential medical implications. We observed that ciliogenesis was profoundly disturbed in human bronchoepithelial cells overexpressing *ZNF676*. Furthermore, a single-nucleotide polymorphism increasing *ZNF676* expression was previously linked to cases of azoospermia [38]. This warrants exploring further whether anomalies in this KZFP or its TE targets might underlie some ciliopathies or defects in male fertility.

## Conclusions

We identify hominoid-specific ZNF676 as a repressor of LTR12C elements and regulatory regions seeded by this TE family into transcriptional networks important in early development and the germline. Its closest paralog ZNF728 has evolved to become a ligand of the full-length provirus ERV9 associated with LTR12 elements. These evolutionarily recent KZFPs emerged concomitantly with their LTR12/ERV9 targets during hominoid evolution. Binding of ZNF676 is associated with protection of targeted LTR12C elements from genome-wide demethylation during PGC development, and highly conserved genes from a transcriptional network related to ciliogenesis and flagellogenesis have been rewired to become regulated by LTR12C and ZNF676.

## Materials and methods

### Data sources

RNA-sequencing reads were downloaded from Gene Expression Omnibus or ArrayExpress (references GSE36552, GSE72379, GSE59435, E-MTAB-2857 were used). Human PGC RNA-seq was obtained from NCBI SRA with the reference SRP057098. GTEx consortium RNA-seq data was obtained from dbGaP Study Accession phs000424.v7.p2. ChIP-seq data was downloaded from GSE75868 and from https://www.encodeproject.org/. ENCODE project datasets that were used are listed in Supplementary Table 5. RRBS methylation of human PGC development was downloaded from Gene Expression Omnibus at reference GSE63818.

### Mapping

Reads were mapped to the human (hg19) genome using Hisat v2.1.0 [42] with parameters hisat2 -k 5 --seed 42 p 7. For stranded data, the --rna-strandness RF parameter was added.

### Summary counts

Counts on genes and transposable elements (TEs) were generated using featureCounts [46]. To avoid read assignation ambiguity between genes and TEs, a .gtf file containing both was provided to featureCounts. For repetitive sequences, an in-house curated version of the Repbase database was used (fragmented LTR and internal segments belonging to a single integrant were merged, see below). Only uniquely mapped reads were used for counting on genes and TE integrants. TEs overlapping exons were discarded from the analysis. Finally, the features for which the sum of reads across all samples was lower than the number of samples were discarded from the analysis.

### Normalization

Normalization for sequencing depth was performed for both genes and TEs using the TMM method as implemented in the limma package [66] of Bioconductor [25] and using the counts on genes as library size.

### Differential expression

Differential gene expression analysis was performed using voom [44] as implemented in the limma package of Bioconductor. A moderated t-test (as implemented in the limma package [66]) was used to test significance. P-values were corrected for multiple testing using the Benjamini-Hochberg method [3]. A gene (or TE) was considered to be differentially expressed when the fold change between groups was higher than 2 and the adjusted p-value was smaller than 0.05.

### Sum on TE subfamilies

For counting on TE subfamilies, reads on the TE integrants of each subfamily were summed without filtering multi-mapped loci out.

### Repeat library merging

Briefly, annotated LTR elements from a list corresponding to the hg19 genome generated by RepeatMasker 4.0.5 (Repeat Library 20140131), when found next to a full-length ERV, were merged with the corresponding -int to form a single genomic feature, as described [83]. Fragmented features were also reassembled. This list was used as reference for transposable elements coordinates for most analyses in this work unless specified otherwise.

### Multiple sequence alignment plot

FASTA sequences for HERV9, HERV9N, HERV9NC, LTR12, LTR12_, LTR12B, LTR12C, LTR12D, LTR12E loci were extracted from the hg19 genome assembly using the bedtools getfasta tool [65]. LTR12C loci were also 5’ extended by 500 bp. Sequences belonging to each individual TE family were then aligned using MAFFT [40] with parameters --reorder --auto. Multiple alignments for HERV9, HERV9N and HERV9NC were then merged together using MAFFT’s -merge option, and so were the alignments for all 6 LTR12 subfamilies. Regions in the alignments consisting of more than 85% of gaps were trimmed out. LTR12 alignments were plotted using Aliview colors, and for each aligned integrant among all three HERV9 and the LTR12C subfamilies, the ZNF676, ZNF728 and KAP1 ChIP-seq signal was extracted from the .bam alignment files using the python pysam library and scaled to the [0,1] interval before being superimposed on the alignments. Finally, average ChIP-seq signals across the aligned integrants were plotted on top. Proviral genomic features were labeled as detected by RetroTector [71, 72] on the consensus for ERV9 elements in the case of HERV9, HERV9N, and HERV9NC, or as previously described [49] in the case of LTR12C.

### Transpochimeric transcripts analysis

A per sample transcriptome was first computed from the embryo [88], oocyte [31] and GTex [50] RNA-seq .bam files using Stringtie [60] with parameters –j 1 –c 1. Each transcriptome was then intersected using BEDTools [65] with both the ENSEMBL hg19 coding exons and curated RepeatMasker output to extract transpochimeric gene transcripts (TcGT) for each sample. Second, a custom Python program was used to annotate and aggregate the sample level TcGTs into counts per groups (development stage or tissue). Briefly, for each dataset, a .gtf file containing all TcGTs was created and then filtered to only keep transcripts with their TSS in an LTR. From this filtered file, TcGTs were annotated for their cognate genes and aggregated when a TSS was located within 100 bp of another TcGT annotated for the same gene. Finally, for each TcGT aggregate, its occurrence per group (tissues, cell type, developmental stage) was computed and added up per TE subfamily. Enrichments per TE subfamily were inferred using a hypergeometric test and the log10 adjusted [3] p-values and TcGT counts were plotted as a heatmap using python.

### TE classification into patterns

To establish groups corresponding to expression patterns, TEs were clustered according to their normalized expression values (z-scores) across samples using correlation as distance in the hclust R function. The cutree R function was then used to extract the 2 main groups.

### Gene ontology

Genes with a TSS within 50 kb of LTR12C elements were selected for gene ontology analysis. We looked for over-representation of GO terms from the Cellular Component (CC) group in these gene sets using the GOstats R package [19] and its embedded hypergeometric test. For plotting, the top 20 significant GO terms as determined by the p-value were used. Enriched terms containing less than 5 genes in total were excluded from the analysis. For genes close to LTR12C elements located in the maternal cluster, gene lists included in each term were retrieved.

### Cell culture

HEK293T cells were cultured in DMEM (Gibco, 41966029) supplemented with 10% FCS and 1% penicillin/streptomycin/glutamine and passaged with Trypsin-EDTA (Gibco, 25200056).

H1 hESCs (WA01) were purchased from WiCell and cultured on matrigel (BD, 354277) in mTESR1 medium (StemCell, 85850). They were passaged using TrypLE (Gibco, 12604021) and the medium was supplemented with 10 μM Y-27632 (abcam, ab120129) during the first 24 hours after splitting.

K562 were purchased from ATCC and cultured in RPMI 1640 (Gibco, 21875034) supplemented with 10% FCS and 1% penicillin/streptomycin/glutamine. They were passaged by dilution and dead cells were removed with Ficoll-Paque (GE Healthcare, 45-001-750).

Win1 naive hESCs were obtained from the Whitehead Institute [80] and cultured in 4i/L/A on gelatin (Millipore, ES-006-B) and DR4+ MEF feeders. MEF feeders were produced from irradiation-inactivated passage 1 DR4+ mouse embryonic fibroblasts cultured in DMEM with 15% FCS, 1% penicillin/streptomycin/glutamine (Thermo Fisher, 10378016), 1x non essential amino acids (Thermo Fisher, 11140035), 1 mM sodium pyruvate (Sigma, S8636) and 0.1 mM β-mercaptoethanol (Thermo Fisher, 31350010).

NHBE cells were purchased from Lonza (CC-2540S) and cultured in PneumaCult EX Plus (StemCell, 05040) during the expansion phase in a T25 flask, according to manufacturer’s instructions. Cells were then passaged into 6.5 mm Transwell inserts with a 0.4 um pore size (Corning, 3470) at a density of 100’000 cells/cm^2^ with 100 μl and 500 μl PneumaCult EX Plus in the apical and basal chambers respectively for further expansion. Upon confluence, medium in the apical chamber was removed and medium in the basal chamber was replaced by PneumaCult ALI (StemCell, 05001) reconstituted according to manufacturer’s instructions for differentiation at an air-liquid interface. Cells were washed with D-PBS weekly to avoid mucus accumulation. After 28 days of differentiation, cells were collected for analysis.

Medium was replaced every two days in either culture phase.

## Plasmid construction and cloning

### shRNA constructs

TRC shRNA oligos [54] targeting ZNF676 (TRCN0000107578) were selected for ZNF676 downregulation (5’-CCGGGTCATCCTCAACTGTTAGTTACTCGAGTAACTAACAGTTGAGGATGACTTTTTG-3’, 5’-AATTCAAAAAGTCATCCTCAACTGTTAGTTACTCGAGTAACTAACAGTTGAGGATGAC-3’).

A scrambled shRNA (shScramble) was used as a control (5’- CCGGTCCTAAGGTTAAGTCGCCCTCGCTCTAGCGAGGGCGACTTAACCTTAGG-3’, 5’- AATTCCTAAGGTTAAGTCGCCCTCGCTAGAGCGAGGGCGACTTAACCTTAGGA-3’). After annealing, the oligos were ligated into pLKO.1 (Addgene, #10878) digested with AgeI and EcoRI.

### pTRE, pRRL overexpression constructs

Constructs coding for LacZ, ZNF728 and the reconstructed full-length form of ZNF676 were codon-optimized for expression in Homo Sapiens, synthesized without a stop codon (GeneArt, Thermo Fisher Scientific) and cloned into pDONR221. They were subsequently shuttled into the destination pTRE-3HA-puro dox-inducible expression vector through an LR-Gateway reaction. The HA-tagged sequences were then amplified (5’- TCAACAAGTTTGTACATTCGAGCTCCGTCATCAACAAGT-3’, 5’- TGGGCGGCCGCGTTTCGCTAGCACGCGCGAGCT-3’) for cloning into the pRRL-IRES-Blast vector for hESC expression. pRRL-IRES-Blast was digested with BsrGI/PmeI and the PCR products were recombined into the digested backbone through an InFusion reaction (Takara, 639650) according to manufacturer’s protocol.

### Lentiviral vector production and transduction

Lentiviral particles for pLKO, pTRE and pRRL vectors were produced in HEK293T and titrated as described [1] for addition to the cell medium.

Win1 naive hESCs were transduced with shRNA vectors at a multiplicity of infection of 10. Downregulation of ZNF676 mRNA was verified by RT-qPCR. HEK293T and H1 were transduced at a multiplicity of infection of 10.

### ChIP-exo and ChIP-seq

HEK293T and H1 hESCs stably expressing HA-tagged ZNF676 and LacZ cloned into the pTRE-3HA-puro or the pRRL-IRES-Blast vector respectively were used.

Expression was confirmed through Western blot. 30 million cells per IP were collected and the chromatin was prepared and sonicated as described [35]. ZNF676 ChIP-seq was performed in biological triplicates.

ChIP-exo samples both for ZNF676 and ZNF728 were processed as described and sequenced on an Illumina HiSeq 2500 to 15 million 100 bp single-end reads.

ChIP-seq samples were lysed and sonicated as described [35]. Total input samples (one per cell line) were collected. The IP was then performed with the anti-HA.11 antibody (clone 16B12, Covance, MMS-101P) incubated overnight at 4°C on a rotating wheel, and captured using protein G Dynabeads (Thermo Fisher, 10004D). Samples then underwent 5-minute washes on a rotating wheel: twice in low salt buffer (10 mM Tris-HCl pH 8, 150 mM NaCl, 1mM EDTA, 1% Triton X-100, 0.15% SDS, 1x PMSF), once in high salt buffer (10 mM Tris-HCl pH 8, 500 mM NaCl, 1 mM EDTA, 1% Triton X-100, 0.15% SDS, 1x PMSF), once in LiCl wash buffer (10 mM Tris-HCl pH 8, 1 mM EDTA, 0.5 mM EGTA, 250 mM LiCl, 1% NP40, 1% NaDOC, 1x PMSF). TE buffer was then added to the beads and the supernatant was removed.

Beads were resuspended in elution buffer (TE buffer, 1% SDS, 150 mM NaCl), treated with Proteinase K (Thermo Fisher, AM2548) and decrosslinked overnight at 65°C and 1100 rpm. The DNA was then purified using a MinElute PCR Purification kit (Qiagen, 28004).

Illumina adapters were ligated to libraries. ChIP-seq samples were pooled and sequenced on an Illumina NextSeq 500 instrument to approximately 40 million paired-ends read per sample.

### ChIP-seq analysis

A previously published KAP1 ChIP-seq performed in WIBR3 human naive embryonic stem cells [79] was downloaded from GSE75868 and processed alongside in-house produced ChIP-seq and ChIP-exo.

Reads were de-multipexed and mapped to the human genome (hg19) using the short read aligner program Bowtie2, using the sensitive local mode (exact parameters: bowtie2 -p 6 -t --sensitive-local -x).

In the case of ZNF676 ChIP-seq performed in H1 hESC, PCR duplicates were tagged with the MarkDuplicates Picard tool (http://broadinstitute.github.io/picard/) and removed using samtools 1.9 [45].

Before peak calling, .bam files were filtered, removing low quality (mapq<10) reads as well as reads aligning to ENCODE blacklist regions and to regions of ChIP experiments with high signal in the input using the Bioconductor package GreyListChIP (https://bioconductor.org/packages/GreyListChIP/). KAP1 and ChIP-exo peaks were called using MACS while ZNF676/ZNF728 ChIP-seq peaks were called using MACS2 [21], using the --BAMPE option for paired-end reads. For ChIP-exo samples, a previously derived total input file was used (GSM2466444, GEO Accession Viewer). For ZNF676, peaks from biological replicates were merged using the Bioconductor package DiffBind (https://bioconductor.org/packages/DiffBind/). Only peaks that occurred in at least 2 samples were conserved and merged into consensus peaks.

Processed ChIP-seq data (.bed files with called peaks, Supplementary Table 5) from the ENCODE project were downloaded from https://www.encodeproject.org/ [12, 70].

The intersectBed tool (with default parameters and unique option) from the BEDTools suite [65] was used to calculate intersections, and the BEDTools genomeCoverageBed tool was used to generate coverage files, which were converted into bigWig files with the bedGraphToBigWig tool [41].

### TE subfamily enrichment analysis

Enrichment analyses were performed on ChIP-seq and ChIP-exo data using an exact binomial test on a set of overlaps between the ChIP and a curated TE database as described above (a peak was included when the overlap was above 50%), made with the BEDTools intersect utility [65]. The number of peaks on each TE subfamily was compared to a random distribution while correcting for the total genomic size of each TE family.

### Co-immunoprecipitations

KZFP and KAP1 co-immunoprecipitation was performed as described [30] on 10 million K562 cells overexpressing HA-tagged ZNF676, ZNF728 and LacZ cloned into the pTRE construct. Expression was induced with doxycycline and cells were harvested by pelleting.

### Repression assay

Target loci of ZNF676 and ZNF728 were randomly selected among ChIP-seq identified peaks correlated with KAP1 peaks in naive hESC for PCR amplification and directional TOPO cloning. In the case of LTR12C, a complete LTR was amplified (5’-TCAGATGACCTAAAATGACATTGAC-3’, 5’- CACCAATAGTCACGATGTTTTGTCCG-3’). For ERV9 targets, a ~500 bp area under the ChIP-seq peak was targeted (5’-GAGGGTCCGTGGCTTCATTC-3’, 5’- CACCAGGCTCCCCATATCCTAGCTT-3’). A HERVK14C sequence known not to be spontaneously repressed in HEK293T [83] and not containing any ZNF676 or ZNF728 binding sites was used as a negative control. PCR products were then cloned into the pENTR-D-TOPO construct and subsequently shuttled into the pRRL-GW-PGK-GFP destination vector through an LR-Gateway reaction. Lentiviral particles were produced and used to transduce HEK293T cells with a multiplicity of infection of 0.25. GFP-positive cells were then isolated through FACS sorting and transduced with dox-inducible constructs coding for HA-tagged KZFPs or LacZ. KZFP expression was induced with doxycyclin 5 days post-transduction, and fluorescence was monitored twice a week starting at transduction and for 22 subsequent days.

### Naive hESC culture and shRNA-mediated knockdown

Win1 naive embryonic stem cells were cultured on irradiated mouse fibroblast feeders in 4i medium as described [80] and passaged with Accutase (Thermo Fisher, A11105-01). After transduction with shRNA-containing pLKO constructs, selection with puromycin was performed. Knockdown levels of ZNF676 were verified by RT-qPCR. The experiment was performed in biological triplicates. Cells were then harvested for RNA-seq by pelleting.

### Identification of paralogous KZFPs in the human and orthologous KZFPs in primate genomes, KZFP aging

KZFPs were annotated as described [35] in human and primate genomes. The “zinc fingerprint” composed of four [87] DNA-binding amino acids per zinc finger and the KRAB domain of human ZNF676 and ZNF728 were then compared to these of all other KZFPs from the human genome and those of other species. For non-human KZFPs, the relatedness to the human KRAB and ZNF domains was assessed through an in-house scoring method, and the KZFP with the highest score for that organism was selected as the most likely ortholog candidate.

### Identification of orthologous TE loci across primate genomes

For all HERV9, HERV9N, HERV9NC and LTR12C loci coordinates identified in human Repbase (hg38), the corresponding coordinates in chimpanzee (panTro6), gorilla (gorGor6), orangutan (ponAbe3), gibbon (nomLeu3), rhesus macaque, crab-eating macaque (macFas5) and olive baboon (chlSab2) genomes were computed with liftOver [32]. The FASTA sequence of every syntenic locus was then retrieved and aligned to the human sequence. Repbase annotation of the primate TE locus, if any, was retrieved. Loci with an annotation divergent from the human one or not annotated as TEs at all were flagged. The percentage of the human sequence that aligned to the foreign species (ignoring point substitutions) was normalised by the human consensus length to correct for short sequences and used as the heatmap color intensity.

### NHBE transduction

Upon plating into Transwells, lentiviral vector was added to the medium of NHBE cells in suspension in the upper chamber for a multiplicity of infection of 5. Transgene expression was induced through addition of doxycycline to the medium in the lower chamber upon transition to the air-liquid interface. Cells were cultured with doxycycline over the course of differentiation.

### Immunofluorescent staining

Differentiated NHBE cells expressing HA-tagged ZNF676 or LacZ were washed with PBS. The Transwell membrane was then cut out and fixed with 4% PFA for 15 minutes, followed by three one-minute PBS washes. The cells were then permeabilized with 0.2% Triton X-100 in PBS, washed three times for one minute with PBS and blocked with 1% BSA in PBS for 45 minutes. Primary antibodies (rat anti-HA, Sigma, 11867423001, 1:1000, mouse anti-acetylated tubulin, Sigma, T7451, 1:250) were diluted in 1% BSA in PBS and the membrane was incubated for 1 hour. Three one-minute PBS washes were performed. The membrane was subsequently incubated with secondary antibodies (goat anti-rat Alexa Fluor 647, A-21247, Thermo Fisher, goat anti-mouse Alexa Fluor, A-11029, Thermo Fisher, both 1:1000) and counterstained with DAPI. After 3 PBS washes, the membranes were mounted on glass slides with Fluoromount-G (Thermo Fisher, 00-4958-02) and imaged using a Nikon Eclipse Ti2-E inverted microscope coupled with a Yokogawa CSU W2 confocal spinning disk unit and equipped with a Prime 95B sCMOS camera (Photometrics). Images were edited for contrast with Fiji.

### Scanning electron microscopy (SEM)

Differentiated NHBE cells grown in Transwells were washed with PBS on the apical side for 30 minutes, then fixed for one hour with 2% paraformaldehyde and 1.25% glutaraldehyde in 0.1M phosphate buffer, pH 7.4. They were then washed in cacodylate buffer (0.1M) and then further fixed for 30 minutes in 0.2% osmium tetroxide in the same buffer. Samples were then dehydrated in a graded alcohol series, dried by passing them through the supercritical point of carbon dioxide (Leica Microsystems CPD300) and attached to aluminium sample holders. Finally, they were coated with a 5 nm layer of gold palladium using a sputter coater (Quorum Technologies, Q300T), and imaged with a field emission scanning electron microscope (Merlin, Zeiss NTS).

### Immunohistochemistry

Detection of DNAH11 (rabbit α-DNAH11, Sigma-Aldrich, HPA037470, diluted 1:20) on human lung samples (provided by SwissHistoTech) was performed using the fully automated Ventana Discovery ULTRA (Roche Diagnostics). All steps were performed on the machine with Ventana solutions. Briefly, dewaxed and rehydrated paraffin sections were pre-treated with heat using extended condition (64 minutes) CC1 solution. The primary antibody was incubated 1 hour at 37°C. After incubation with rabbit Immpress HRP (Ready to use, Vector Laboratories), chromogenic revelation was performed with ChromoMap DAB kit (Roche Diagnostics). Sections were counterstained with Harris haematoxylin and permanently mounted.

### RNA extraction

Win1 RNA was extracted with a High Pure RNA Isolation kit (Roche, 11828665001) according to manufacturer’s instructions. NHBE RNA was extracted using a NucleoSpin RNA XS (Macherey-Nagel, 74090250) as per the manufacturer’s protocol.

### Library preparation and sequencing

Libraries were prepared for RNA sequencing with the TruSeq Stranded mRNA Library Prep kit (Illumina, 20020594).

ChIP-exo libraries were prepared as previously described [35] and ChIP-seq libraries were ligated to Illumina adapters.

NHBE RNA-seq libraries (to a minimum depth of 50 million reads per sample) and ChIP-seq libraries (to a minimum depth of 40 million reads per sample) were sequenced on a NextSeq 500 instrument with paired-end reads of 75 bp.

Win1 RNA-seq libraries were sequenced on an Illumina HiSeq 4000 to a minimum depth of 30 million 100 bp paired-end reads per sample.

ChIP-exo libraries were sequenced on an Illumina HiSeq 2500 to a minimum depth of around 15 million 100 bp single-end reads.

### hPGC DNA methylation analysis

RRBS methylation data was downloaded from GSE63818. CpG data was extracted and intersected with Repbase (20140131) in order to identify CpGs contained within individual TE loci. Methylation levels on these CpGs were averaged to obtain an average methylation level per TE locus. Loci without any profiled CpGs were excluded from the analysis. Replicate values were averaged where available. The subfamily methylation level was computed by averaging values for all detected member loci.

## Supplementary Information

**Additional file 1: Figure S1.** (A) Number of detected LTR-driven TcGTs in a de novo transcriptome assembly of bulk RNA-seq of human oocytes [31], method same as in Fig. 1A. GV = germinal vesicle, MI = meiosis I, MII = meiosis II. (B) Z-score clusters of expression of all detected LTR12/ERV9 loci over human embryonic development stages. Blue = LTR12C loci with at least one detected TcGT at any stage of development, grey = no known TcGT. (C) Examples of junction coverage in LTR12C-initiated TcGTs among those identified in Fig. 1A as found in single-cell oocyte sample GSM896803. TEs initiating transcription highlighted in pink. **Figure S2.** (A) Heatmap displaying KAP1, NFYA/B ChIP-seq & KZFP ChIP-exo enrichment over all TE subfamilies which present a binomial p-value above 0.05 for at least one of the profiled factors. NFYA/B ChIP-seq from ENCODE. KAP1 ChIP-seq in naive hESCs from [79]. p-value obtained by binomial test, corrected for TE subfamily size. (B) Heatmap displaying enrichment of all biological replicates and consensus enrichments of ChIP-seq and ChIP-exo performed for ZNF676 and ZNF728. Statistics as in (A). Right, overlap between peaks on LTR12C and KAP1 peaks in naive hESC on LTR12C (bedtools). (C) ZNF676 and ZNF728 expression in reset to naive and primed H9 hESCs, RNA-seq from Takashima et al. [76]. (D) Sashimi plots of ZNF676 transcripts and splicing patterns showing the missing exon observed in representative examples of 8-cell and morula stage embryos [88], testis tissue from GTex consortium (gtexportal.org), and H9 hESCs reset to the naive state [76]. Transcript track for hg19, RefSeq. (E) Multiple sequence alignment (Clustal Omega) of Uniprot-annotated ZNF676 and ZNF728 protein sequence alongside translated reconstructed ZNF676 following the splicing patterns from (D). Reconstructed ZNF676 was used for all experiments involving ovexpression. KRAB domain as detected by Uniprot highlighted in red. (F) Scheme of lentiviral vectors with KZFP targets used for repression assay. TE fragments are cloned in antisense direction. (G) Enrichment for KAP1-bound TE integrants within 50 kb of the TSS of genes upregulated upon ZNF676 shRNA-mediated knockdown in Win1 naive embryonic stem cells. Binomial test. **Figure S3.** (A) Spread of human solo LTR12C integrants and syntenic loci across primate genomes. Heatmap depicting all Repeatmasker-annotated LTR12C loci in hg38 human genome and their liftOver orthologous loci in primate species. White = not detected. Red = detected and annotated as same subfamily member. Blue = detected and annotated as same family member. Grey = detected and not annotated. Color intensity = similarity (by percentage of sequence aligned) normalized by integrant length relative to consensus. **Figure S4.** (A) Heatmap of z-score expression of ZNF676 and other KZFPs across hPGC development. RNA-seq data from [77]. (B) ZNF676 and ZNF728 expression across GTex consortium tissues, data from gtexportal.org. (C) Z-score clusters of expression of all detected LTR12C loci over human embryonic development stages [88], as identified in Fig. 1C. Purple lines = LTR12C loci with ZNF676 binding (ChIP-seq in HEK293T cells). Grey lines = LTR12C loci without ZNF676 binding. Thick black line: mean expression value across replicates for each sample. **Figure S5.** (A) Gene Ontology Cellular Compartment terms for which genes within 50 kb of an LTR12C expressed in testis are enriched. Top 20 terms, terms smaller than 5 genes excluded. (B) Dysregulation of cilium-related genes upon ZNF676 knockdown in human naive embryonic stem cells (Win1). DNAH11 bears an LTR12C at its annotated promoter. C4orf47 has a ZNF676 binding site overlapping with a KAP1 binding site at its TSS. (C) IHC staining of human bronchial samples by anti-DNAH11 antibody (HPA045880, Sigma). Nuclei stained with hematoxylin. (D) Examples of putative LTR12C/ZNF676-connected genes related to the cilium/flagellum identified in our study.

**Additional file 2: Supplementary Table 1.** List of identified LTR12C-initiated TcGTs across human embryonic development.

**Additional file 3: Supplementary Table 2.** Top 20 Cellular Component Gene Ontology Terms (by p-value computed by the GO Stats R package) identified across genes located within 50kb of LTR12C from the maternal cluster.

**Additional file 4: Supplementary Table 3.** Top 20 Cellular Component Gene Ontology Terms (by p-value computed by the GO Stats R package) identified across genes located within 50kb of LTR12C expressed in testis.

**Additional file 5: Supplementary Table 4.** Top 20 Cellular Component Gene Ontology Terms (by p-value computed by the GO Stats R package) identified across genes differentially expressed between ZNF676-overexpressing and LacZ-overexpressing differentiated NHBE cells.

**Additional file 6: Supplementary Table 5.** List of ENCODE ChIP-seq data files downloaded and analyzed in this work.

## Data Availability

All high-throughput sequencing data generated during this study have been deposited into NCBI Gene Expression Omnibus (GEO) with reference GSE163580, accessible with token *cnotekoyzlqlvel* before publication. RNA-seq experiments include .txt files with counts on genes and TEs, while ChIP-seq and ChIP-exo experiments include .bed files with peaks called by MACS or MACS2.0.
