# Supplementary material for: KRAB zinc finger protein ZNF676 controls the transcriptional influence of LTR12-related endogenous retrovirus sequences
Source: Mob DNA. 2022 Jan 18;13:4. doi: 10.1186/s13100-021-00260-0 (PMC8767690; doi:10.1186/s13100-021-00260-0)
Supplement: Supplementary file 1 — Additional file 1: Figure S1. (A) Number of detected LTR-driven TcGTs in a de novo transcriptome assembly of bulk RNA-seq of human oocytes [31], method same as in Fig. 1A. GV = germinal vesicle, MI = meiosis I, MII = meiosis II. (B) Z-score clusters of expression of all detected LTR12/ERV9 loci over human embryonic development stages. Blue = LTR12C loci with at least one detected TcGT at any stage of development, grey = no known TcGT. (C) Examples of junction coverage in LTR12C-initiated TcGTs among those identified in Fig. 1A as found in single-cell oocyte sample GSM896803. TEs initiating transcription highlighted in pink. Figure S2. (A) Heatmap displaying KAP1, NFYA/B ChIP-seq & KZFP ChIP-exo enrichment over all TE subfamilies which present a binomial p-value above 0.05 for at least one of the profiled factors. NFYA/B ChIP-seq from ENCODE. KAP1 ChIP-seq in naive hESCs from [79]. p-value obtained by binomial test, corrected for TE subfamily size. (B) Heatmap displaying enrichment of all biological replicates and consensus enrichments of ChIP-seq and ChIP-exo performed for ZNF676 and ZNF728. Statistics as in (A). Right, overlap between peaks on LTR12C and KAP1 peaks in naive hESC on LTR12C (bedtools). (C) ZNF676 and ZNF728 expression in reset to naive and primed H9 hESCs, RNA-seq from Takashima et al. [76]. (D) Sashimi plots of ZNF676 transcripts and splicing patterns showing the missing exon observed in representative examples of 8-cell and morula stage embryos [88], testis tissue from GTex consortium (gtexportal.org), and H9 hESCs reset to the naive state [76]. Transcript track for hg19, RefSeq. (E) Multiple sequence alignment (Clustal Omega) of Uniprot-annotated ZNF676 and ZNF728 protein sequence alongside translated reconstructed ZNF676 following the splicing patterns from (D). Reconstructed ZNF676 was used for all experiments involving ovexpression. KRAB domain as detected by Uniprot highlighted in red. (F) Scheme of lentiviral vectors with KZFP targets used fo [file 13100_2021_260_MOESM1_ESM.pdf]

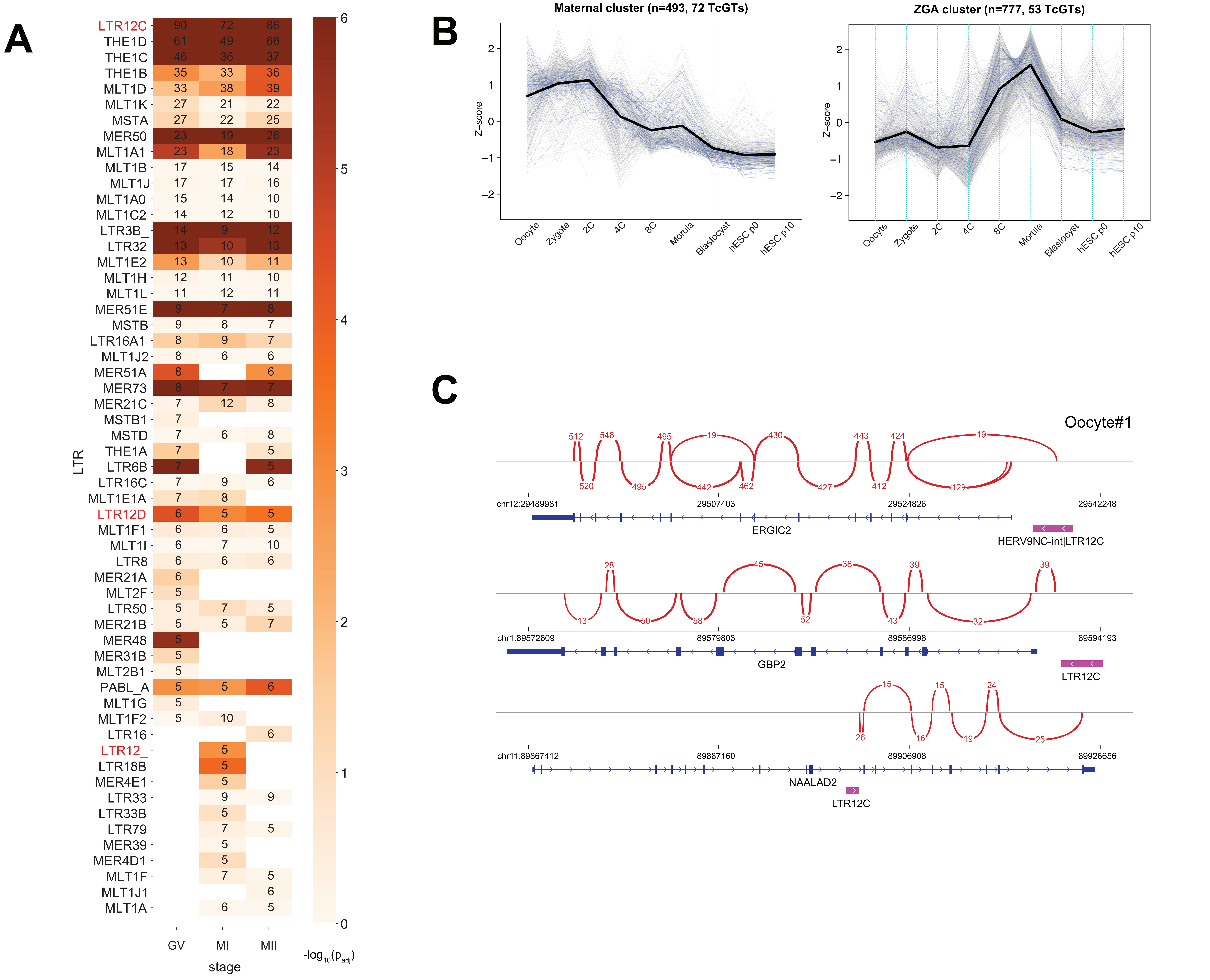

Figure S1 (**A**) Number of detected LTR-driven TcGTs in a de novo transcriptome assembly of bulk RNA-seq of human oocytes (Hendrickson et al., 2017), method same as in Fig.1A. GV = germinal vesicle, MI = meiosis I, MII = meiosis II. (**B**) Z-score clusters of expression of all detected LTR12/ERV9 loci over human embryonic development stages. Blue = LTR12C loci with at least one detected TcGT at any stage of development, grey = no known TcGT. (**C**) Examples of junction coverage in LTR12C-initiated TcGTs among those identified in Fig.1A as found in single-cell oocyte sample GSM896803. TEs initiating transcription highlighted in pink.

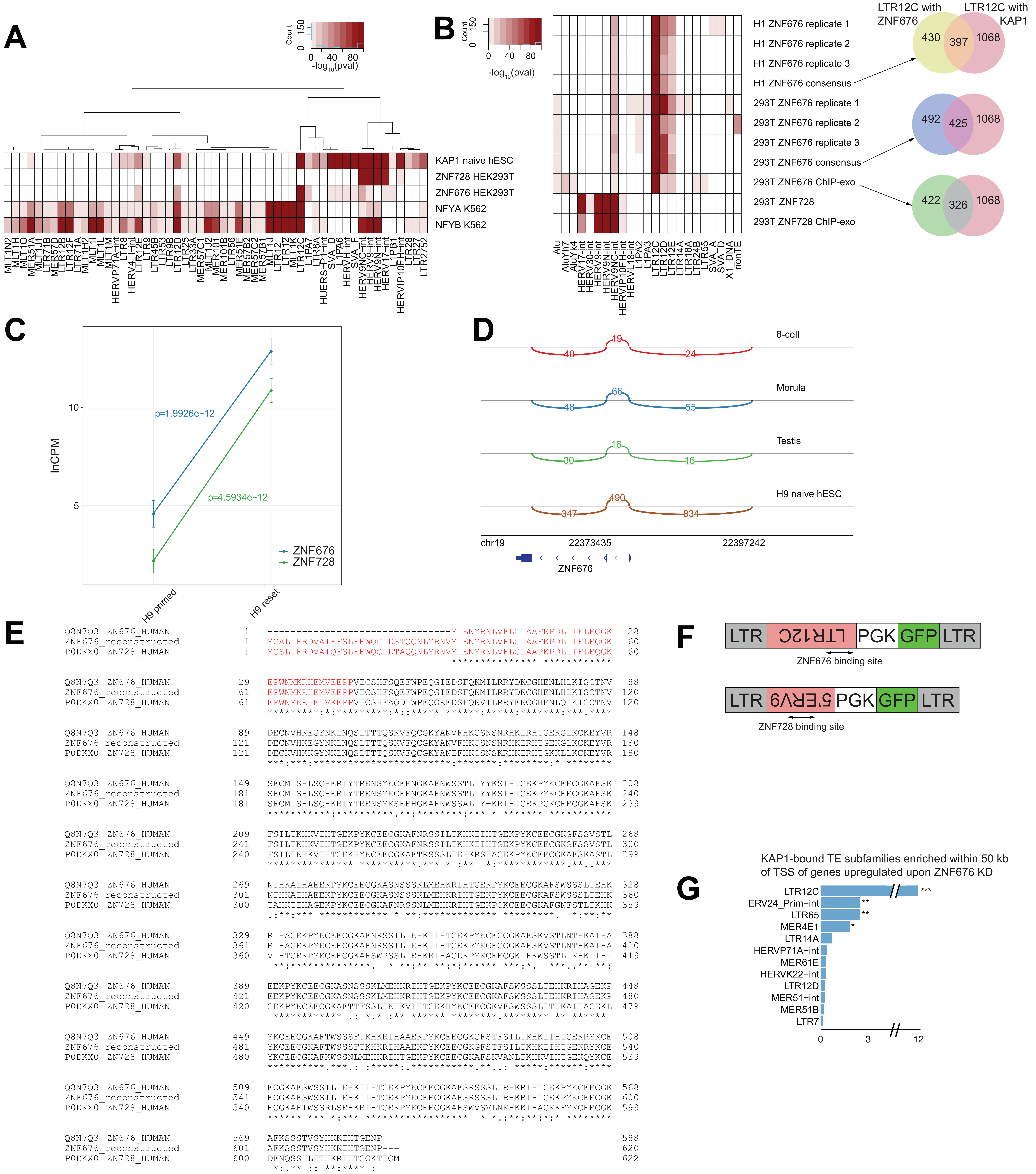

Figure S2 (**A**) Heatmap displaying KAP1, NFYA/B ChIP-seq & KZFP ChIP-exo enrichment over all TE subfamilies which present a binomial p-value above 0.05 for at least one of the profiled factors. NFYA/B ChIP-seq from ENCODE. KAP1 ChIP-seq in naive hESCs from Theunissen et al., 2016. p-value obtained by binomial test, corrected for TE subfamily size. (**B**) Heatmap displaying enrichment of all biological replicates and consensus enrichments of ChIP-seq and ChIP-exo performed for ZNF676 and ZNF728. Statistics as in (A). Right, overlap between peaks on LTR12C and KAP1 peaks in naive hESC on LTR12C (bedtools). (**C**) *ZNF676* and *ZNF728* expression in reset to naive and primed H9 hESCs, RNA-seq from Takashima et al., 2014. (**D**) Sashimi plots of *ZNF676* transcripts and splicing patterns showing the missing exon observed in representative examples of 8-cell and morula stage embryos (Yan et al., 2013), testis tissue from GTex consortium (gtexportal.org), and H9 hESCs reset to the naive state (Takashima et al., 2014). Transcript track for hg19, RefSeq. (**E**) Multiple sequence alignment (Clustal Omega) of Uniprot-annotated ZNF676 and ZNF728 protein sequence alongside translated reconstructed *ZNF676* following the splicing patterns from (D). Reconstructed *ZNF676* was used for all experiments involving overexpression. KRAB domain as detected by Uniprot highlighted in red. (**F**) Scheme of lentiviral vectors with KZFP targets used for repression assay. TE fragments are cloned in antisense direction. (**G**) Enrichment for KAP1-bound TE integrants within 50 kb of the TSS of genes upregulated upon ZNF676 shRNA-mediated knockdown in Win1 naive embryonic stem cells. Binomial test.

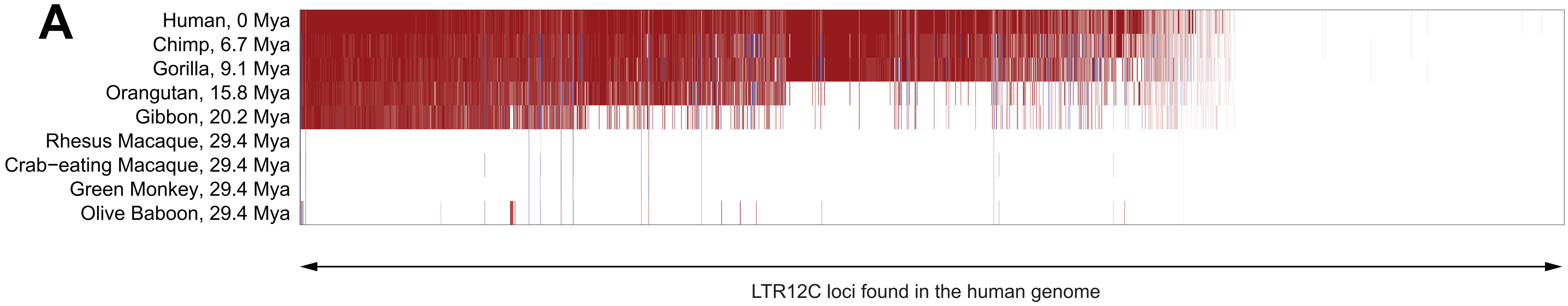

Figure S3 (**A**) Spread of human solo LTR12C integrants and syntenic loci across primate genomes. Heatmap depicting all Repeatmasker-annotated LTR12C loci in hg38 human genome and their liftOver orthologous loci in primate species. White = not detected. Red = detected and annotated as same subfamily member. Blue = detected and annotated. Grey = detected and not annotated. Color intensity = similarity (by percentage of sequence aligned) normalized by integrant length relative to consensus.

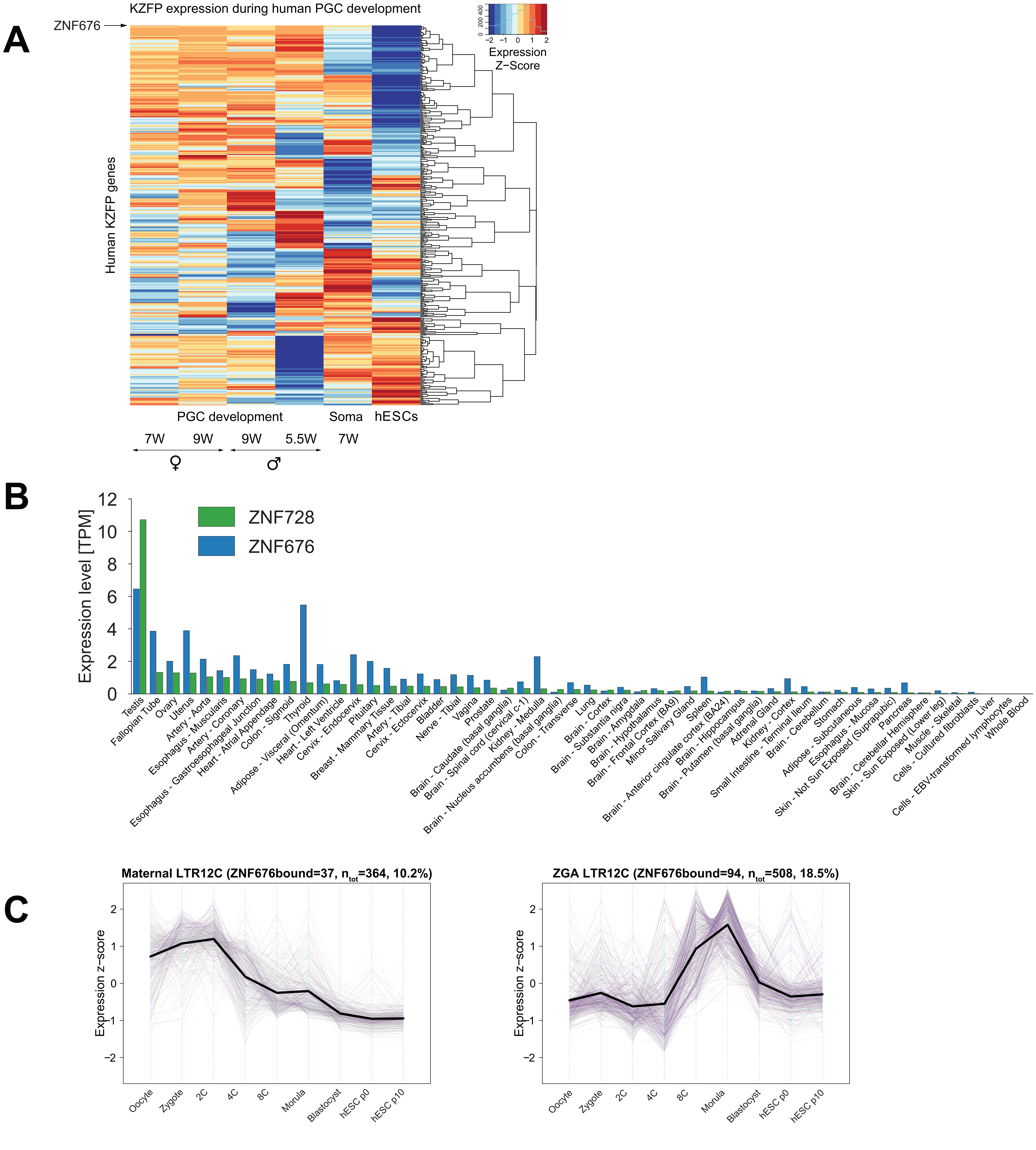

Figure S4 (**A**) Heatmap of z-score expression of *ZNF676* and other KZFPs across hPGC development. RNA-seq data from Tang et al., 2015. (**B**) *ZNF676* and *ZNF728* expression across GTex consortium tissues, data from gtexportal.org. (**C**) Z-score clusters of expression of all detected LTR12C loci over human embryonic development stages (Yan et al., 2013), as identified in Fig.1C. Purple lines = LTR12C loci with ZNF676 binding (ChIP-seq in HEK293T cells). Grey lines = LTR12C loci without ZNF676 binding. Thick black line: mean expression value across replicates for each sample.

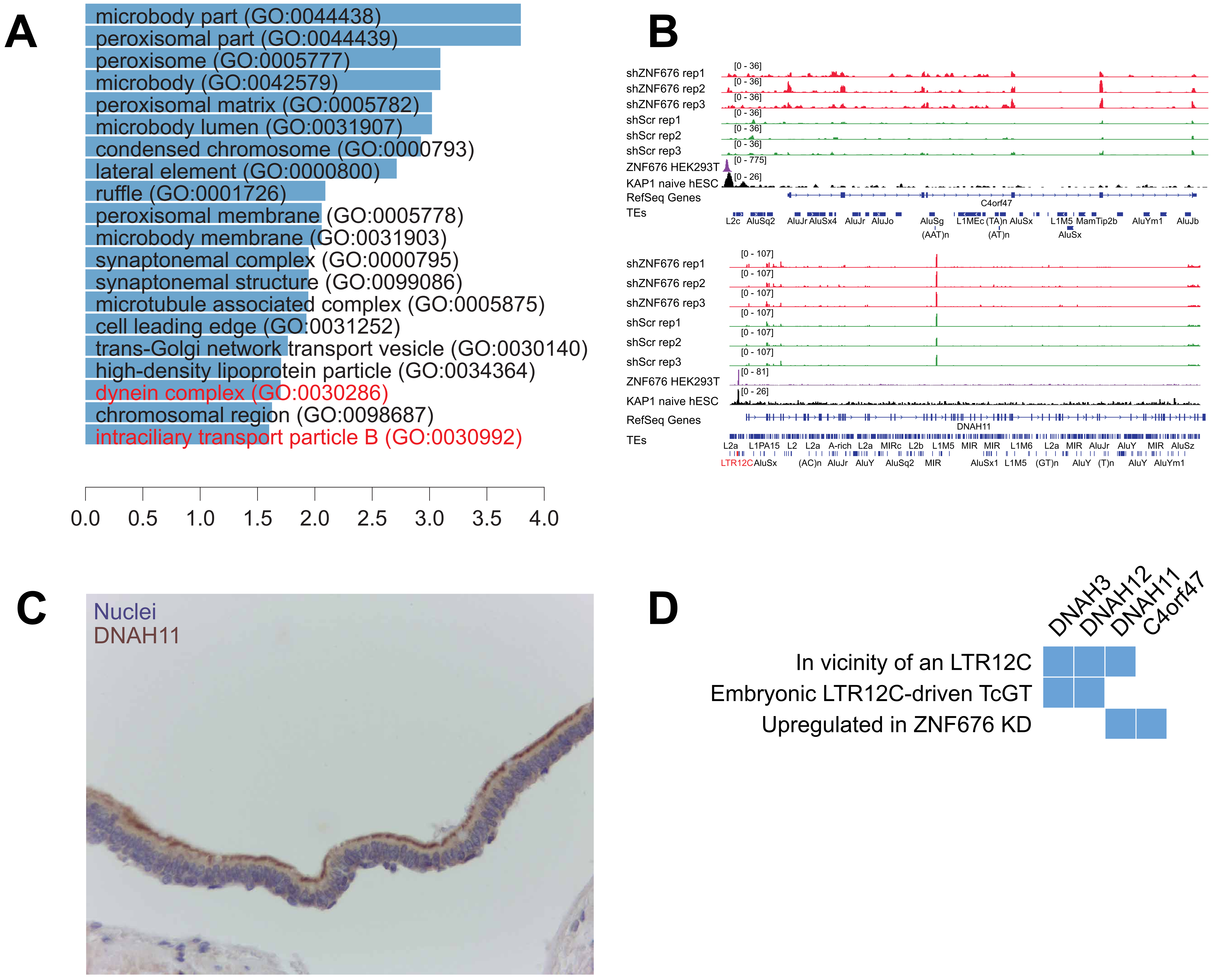

Figure S5 ZNF676 regulates several germline genes involved in ciliary motility, and its overexpression is able to disrupt ciliogenesis. (**A**) Gene Ontology Cellular Compartment terms for which genes within 50 kb of an LTR12C expressed in testis are enriched. Top 20 terms, terms smaller than 5 genes excluded. (**B**) Dysregulation of cilium-related genes upon *ZNF676* knockdown in human naive embryonic stem cells (Win1). *DNAH11* bears an LTR12C at its annotated promoter. *C4orf47* has a ZNF676 binding site overlapping with a KAP1 binding site at its TSS. (**C**) IHC staining of human bronchial samples by anti-DNAH11 antibody (HPA045880, Sigma). Nuclei stained with hematoxylin. (**D**) Examples of putative LTR12C/ZNF676-connected genes related to the cilium/flagellum identified in our study.
